# Stable and low-threshold photon upconversion in nondegassed water by organic crystals

**DOI:** 10.3389/fchem.2023.1217260

**Published:** 2023-07-13

**Authors:** Yoichi Murakami, Riku Enomoto

**Affiliations:** Laboratory for Zero-Carbon Energy, Institute of Innovative Research, Tokyo Institute of Technology, Meguro, Tokyo, Japan

**Keywords:** triplet-triplet annihilation, upconversion, organic crystal, solid solution, *in vivo* application

## Abstract

Photon upconversion (UC) is a technology that converts lower-energy photons (longer wavelength light) into higher-energy photons (shorter wavelength light), the opposite of fluorescence. Thus, UC is expected to open a vast domain of photonic applications that are not otherwise possible. Recently, UC by triplet**−**triplet annihilation (TTA) between organic molecules has been studied because of its applicability to low-intensity light, although the majority of such studies have focused on liquid samples in the form of organic solvent solutions. To broaden the range of applications, solid-state UC materials have been an active area of research. We recently developed air-stable, high-performance molecular UC crystals that utilize a stable solid-solution phase of bicomponent organic crystals. This article begins with a brief overview of previous challenges in developing and improving solid-state TTA–UC materials. Then, we briefly review and explain the concept as well as advantages of our molecular solid-solution UC crystals. We applied these organic crystals for the first time to a water environment. We observed blue UC emission upon photoexcitation at 542 nm (green–yellow light) and then measured the excitation intensity dependence as well as the temporal stability of the UC emission in air-saturated water. In nondegassed water, these organic crystals were stable, functioned with a low excitation threshold intensity of a few milliwatts per square centimeter, and exhibited high photo-irradiation durability at least over 40 h; indicating that the developed organic crystals are also viable for aqueous conditions. Therefore, the organic crystals presented in this report are expected to extend the domain of UC-based photonic applications in practical water systems including *in vivo* diagnostic, clinical, and therapeutic applications.

## 1 Introduction

The research area of photon upconversion (UC) by triplet**−**triplet annihilation (TTA), termed TTA–UC, has been progressing since some initial works in early 2000s (Keivanidis et al., 2003; Kozlov and Castellano, 2004) until the present date; many review papers have been published (for example, Singh-Rachford and Castellano, 2010; Simon and Weder, 2012; Schulze and Schmidt, 2015; Gray et al., 2018; Seo et al., 2022; Alves et al., 2022). The applicability to low-intensity light sources, including noncoherent sunlight (Baluschev et al., 2006; Schulze and Schmidt, 2015), differentiates TTA–UC technology from previous UC technology consisting of rare-earth-doped inorganic materials that require high-intensity lasers (Auzel, 2004). However, excited triplet states of organic molecules or chromophores used in TTA–UC are quenched when they are contacted by an oxygen molecule (O_2_) (Turro et al., 2009), which further generates highly reactive singlet oxygen that could irreversibly degrade organic molecules. Furthermore, because intermolecular energy transfer in TTA–UC is based on the Dexter mechanism, which only occurs over a short distance (typically less than 1 nm; Turro et al., 2009), TTA–UC is inherently easier to implement in liquid systems in which the chromophores’ Brownian motion facilitates mutual collisions and hence triplet energy transfer (TET) as well as TTA. Therefore, studies of TTA–UC have mostly been conducted by using degassed organic solvents, in which O_2_ molecules have been removed either by freeze–pump–thaw cycles or inert gas bubbling.

Thus, it has been challenging to develop efficient and durable solid-state TTA–UC materials—‘durable’ refers to stability against continuous photo-irradiation and photo-irradiation in the presence of O_2_—compared with conventional degassed and tightly sealed organic-solvent-solution samples. Initial trials aimed at forming solid-state TTA–UC materials did not result in high efficiency and low excitation thresholds, mainly because of the lack of fluidic solvents and hence the Brownian motion of the triplet-energy-carrying species. A typical case among the initial works developed solid samples by embedding palladium octaethylporphyrin (triplet sensitizer) and 9,10-diphenylanthracene (DPA, triplet annihilator) into polyurethane (Singh-Rachford et al., 2009); UC emission was evident only above the glass transition temperature of the polymer matrix, upon which the chromophores started to undergo translational thermal motions necessary for intermolecular TET. Another initial study (Merkel and Dinnocenzo, 2009) doped platinum octaethylporphyrin (PtOEP, triplet sensitizer) and DPA into a poly (methylmethacrylate) film, but the resulting UC efficiency was much less than 1% because of substantial hindrance of intermolecular TET in such rigid materials.

As will be outlined in a subsequent paragraph, much effort has focused on developing high-performance solid-state TTA–UC materials, which are expected to have broad applications. Furthermore, imparting solid-state TTA–UC materials with aqueous functionalities would facilitate innovative *in vivo* photonic applications encompassing vast domains of diagnostic, clinical, and therapeutic applications. Although several *in vivo* compatible TTA–UC materials have been reported (Liu et al., 2013; Park et al., 2018; Seo et al., 2022; Vepris et al., 2022), the corresponding performance (e.g., UC efficiency and excitation threshold intensity) has been limited or low. In particular, durability under continuous photo-irradiation in nondegassed water has not yet been demonstrated unequivocally to our best knowledge.

In Section 2, we briefly review some representative efforts to develop solid-state TTA–UC materials in the context of the present study. In Section 3, we explain our recently developed strategy of utilizing a thermodynamically stable phase of a bicomponent solid-solution that affords molecularly uniform (i.e., aggregation-free), durable, and efficient TTA–UC solids; which are organic crystals formed by van der Waals forces. In Section 4, we present original results that demonstrate the ability of such organic crystals to carry out stable and durable UC under continuous photo-irradiation in nondegassed water.

## 2 Brief overview of previous solidification strategy

Research on high-performance solid-state TTA–UC materials started in the early 2010s, as documented by several review papers (for example, Simon and Weder, 2012; Joarder et al., 2018; Gray et al., 2018; Seo et al., 2022; Alves et al., 2022). Here we briefly overview some of the history in order to clarify the context of this report.

**FIGURE 1:**
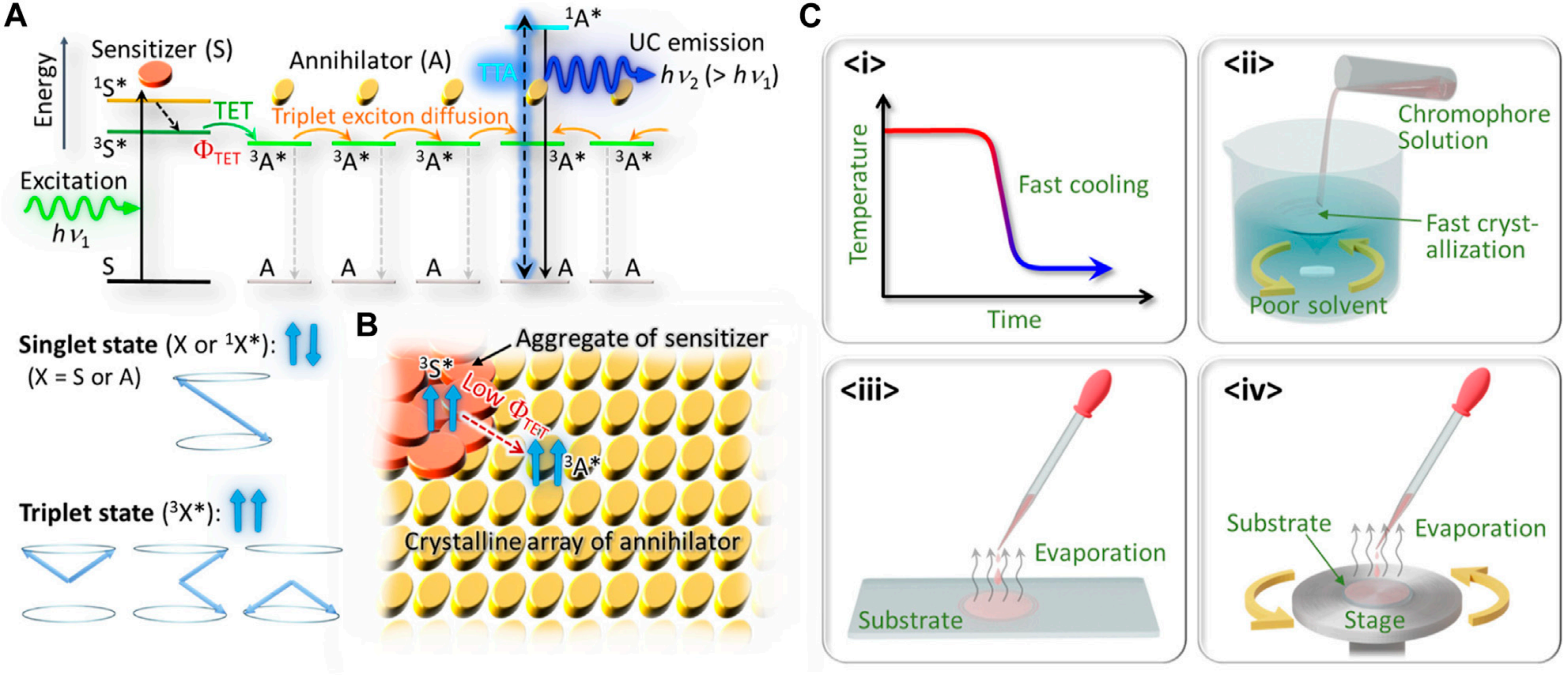
**(A)** Schematic of TTA–UC in solid materials in which lower-energy photons (*hν*_1_) are up-converted into higher-energy photons (*hν*_2_) as a result of triplet energy transfer (TET) and triplet−triplet annihilation (TTA). **(B)** Schematic of sensitizer aggregation on/in a crystal of an annihilator. **(C)** Classification of some typical kinetically controlled approaches taken to date, <i > to <iv>; see main text for details.

Figure 1A indicates the principles of TTA–UC in solid systems. Here, S and A denote the sensitizer and annihilator, respectively; both are usually organic molecules (chromophores). First, S is photo-excited by absorbing a photon of energy of *hν*_1_, which converts S into an excited singlet state (^1^S*). If there is strong intersystem crossing, ^1^S* immediately converts into the excited triplet state (^3^S*), which has much longer lifetime than that of ^1^S*. Furthermore, if there is sufficient orbital overlap between the S and A chromophores, a state of ^3^A* can be created as a result of TET from ^3^S* with a quantum efficiency of Φ_TET_; generally, a low Φ_TET_ might be caused by thermodynamic mismatch between the energy levels of ^3^S* and ^3^A*, too short a lifetime of ^3^S*, or insufficient orbital overlap between the S and A chromophores. Subsequently, the state of ^3^A* might diffuse as a triplet exciton among many A units, until ^3^A* either decays back into the ground state or causes TTA when it encounters another triplet exciton. The latter might cause emission of an upconverted photon of energy *hν*_2_ (>*hν*_1_) from the singlet state of ^1^A* (Figure 1A).

To our knowledge, Monguzzi et al. (2012) is the first explicit study of TTA–UC that used a crystalline organic solid. They tried to dope a PtOEP sensitizer into a crystal of DPA via co-crystallization in the organic solvent, aiming to achieve triplet exciton diffusion through the crystal of DPA. However, because of the substantial aggregation of PtOEP, which formed on the surface of the DPA crystal as a separate phase, TET from PtOEP to DPA was substantially hindered (Figure 1B). As the result, they reported only weak UC emission relative to strong phosphorescence from PtOEP (Monguzzi et al., 2012), an indication of inefficient TET toward DPA. Subsequently, other researchers studied the effect of PtOEP aggregates on the properties of UC emission from a polycrystalline film of DPA (Goudarzi and Keivanidis, 2017).

To overcome this aggregation problem, many researchers have used kinetically controlled strategies, as some of the representative ones are graphically classified in Figure 1C. The first kind (<i> in Figure 1C) is by rapidly cooling a thermal melt of a sensitizer–annihilator mixture to quench it into a homogeneous glassy solid. This method mitigated formation of a sensitizer aggregate but the highly disordered annihilator solid led to low UC performance, such as high excitation threshold intensity (Vadrucci et al., 2014). The second kind (<ii> in Figure 1C) is by rapid precipitation of microcrystals by rapidly mixing the organic solvent solution of chromophores with a poor solvent (Ogawa et al., 2017). Although this method enhanced the dispersibility of the sensitizer in the polycrystalline micropowders of the annihilator, very small (less than 1 μm, by the scanning electron microscope images) crystalline domains in such precipitated powders hindered efficient transport of the triplet excitons in the materials and led to low UC performance. The third kind (<iii> in Figure 1C) used casting of a solution of the sensitizer and annihilator in a low boiling point organic solvent onto a substrate, such that rapid evaporation of the solvent formed a polycrystalline thin film before the sensitizer started to form the aggregate (Hosoyamada et al., 2016; Kamada et al., 2017; Abulikemu et al., 2019). The fourth kind (<iv> in Figure 1C) is similar to <iii> but researchers used spin-coating of an organic solvent solution to form a thin solid film over the entire substrate (Goudarzi and Keivanidis, 2014; Karpicz et al., 2014; Goudarzi and Keivanidis, 2017; Ogawa et al., 2018).

All of these methods mitigated the sensitizer aggregation problem to some extent. However, some researchers have pointed out that “amorphous and polycrystalline thin films of organic semiconductor are characterized by a significant degree of disorder, in particular when they are cast from solution” (Mikhnenko et al., 2015). This statement seems to explain the limited performance of the aforementioned examples, in which the sample fabrication methods relied on fast solidification, which inherently resulted in a substantial thermodynamic nonequilibrium in the samples as well as a high density defects or grain boundaries in such microcrystals. Therefore, mitigating the sensitizer aggregation problem often led to a new problem—limited UC performance—that resulted from the short lifetime and diffusion distance of the triplet excitons in solid materials.

## 3 Organic solid-solution strategy we propose

**FIGURE 2:**
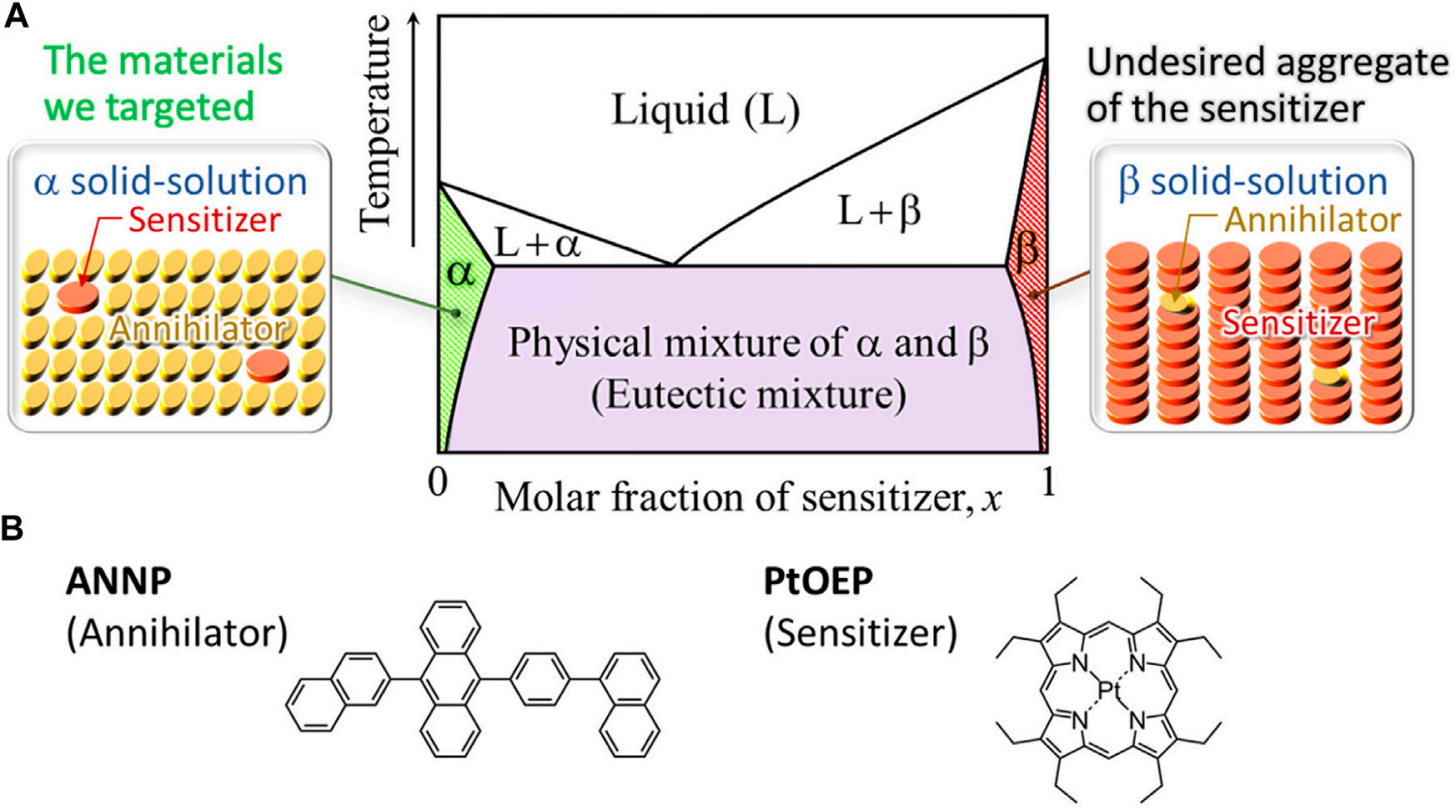
**(A)** Schematic phase diagram representing a bicomponent eutectic system comprised of two solid-solution phases α and β. In present case, an α phase corresponds to an annihilator crystal into which a small quantity of the sensitizer is entropically doped and a β phase is the sensitizer aggregate. The region between the α and β phases represents the eutectic mixture of these phases. **(B)** The annihilator and sensitizer chromophores used in this study.

Recently, we proposed a novel concept in the field of TTA–UC: generating thermodynamically stable bicomponent molecular crystals; we have demonstrated its effectiveness (Enomoto et al., 2021). This concept does not use fast solidification and hence does not result in low crystallinity and small crystalline domains. In particular, we conceived of a solid-solution phase that is generally represented by the α-phase of a bicomponent crystal system, the phase diagram of which is schematically illustrated by Figure 2A. The horizontal position in this diagram represents molar fraction of the sensitizer (*x*; 0 ≤ *x* ≤ 1). By this definition, in the α-phase (*x* ≈ 0), a small quantity of sensitizer is doped into a crystal of the annihilator; this is driven by the increase of the mixing entropy, which is a thermodynamic force that lowers the Gibbs free energy of bicomponent systems (Schroeder, 2000). In the β-phase (*x* ≈ 1), on the contrary, a small quantity of the annihilator is doped into a crystal of the sensitizer; this is the sensitizer aggregate, formation of which should be avoided (Figure 2A). The region between the α- and β-phases is the physical mixture (eutectic mixture) of these phases. Because this eutectic phase contains microaggregates of the sensitizer, formation of this eutectic phase should also be avoided. Therefore, one should opt for the α-phase to achieve the purpose. Accordingly, we searched for optimal conditions that can selectively generate the intended crystals of α-phase by experimentally testing various conditions; including the sensitizer–annihilator combination, type of organic solvent for recrystallization, recrystallization method, temperature, as well as starting concentrations of the sensitizer and annihilator in the solvent.

Eventually, we discovered that the annihilator 9-(2-naphthyl)-10-[4-(1-naphthyl)phenyl]anthracene (ANNP, Figure 2B) can realize our concept. ANNP has two side moieties attached to anthracene, which is a blue-emitting chromophore. These side moieties enable stable accommodation of a small quantity of PtOEP (Enomoto et al., 2021); PtOEP is a common green-absorbing sensitizer in the field of TTA–UC. Such solid-solution-based UC crystals—formed by van der Waals forces—exhibited unprecedented UC performance in air; including a UC quantum efficiency of up to 16% (out of a maximum of 50%), ultralow excitation threshold intensity of ca. 0.2× natural sunlight intensity, and endurance against continuous photoirradiation over tens of hours without photodegradation (Enomoto et al., 2021). However, we did not test whether the van der Waals solid-solution UC crystals were compatible with aqueous environments, despite recent active extension of the application target of TTA–UC into the area of water-compatible (or aqueous) applications (Turshatov et al., 2011; Wohnhaas et al., 2011; Kim et al., 2016; Seo et al., 2022). This is the motivation of the study reported here; we present our experimental results next.

## 4 Results: efficient and durable UC in nondegassed water

**FIGURE 3:**
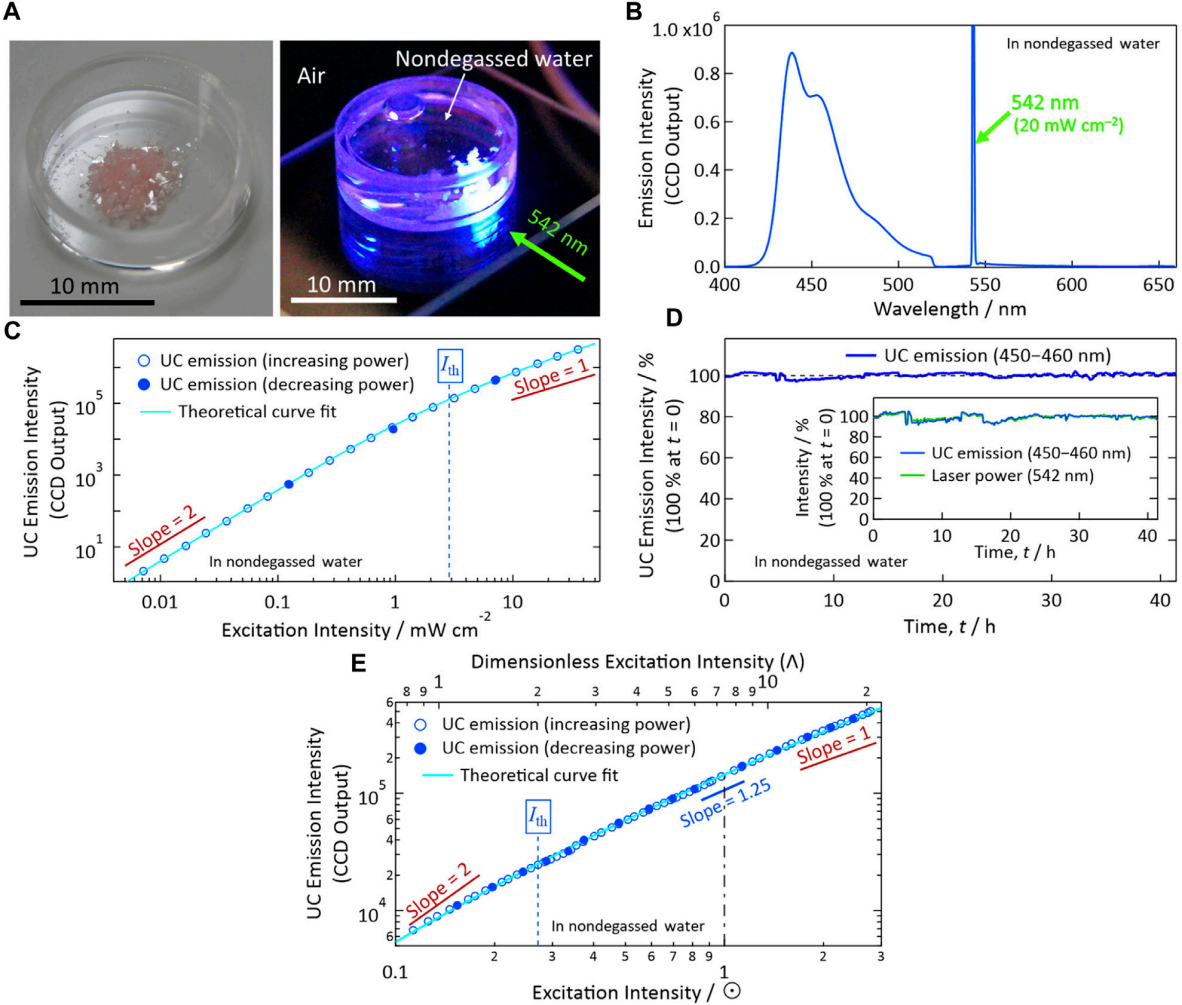
Photoemission properties in nondegassed water. **(A)** Left: Photograph of the solid-solution UC crystals (PtOEP-doped ANNP crystals) used in this report. Note the transparent and slightly pinkish color of the crystals with planer shape. Right: Photograph of the crystals in nondegassed water up-converting an incident laser light at 542 nm (green–yellow) into blue emission. We acquired this photograph through a notch filter to eliminate the incident laser light. **(B)** Photoemission spectrum upon excitation with a 542-nm laser at 20 mW cm^−2^. **(C)** Excitation intensity dependence of the UC emission intensity measured using a 542-nm laser. *I*_th_ indicates the excitation threshold intensity. **(D)** Temporal stability of the UC emission intensity (100% at *t* = 0, spectrally integrated between 450 and 460 nm) under continuous photoexcitation with a 542-nm laser at an intensity of ca. 20 mW cm^−2^. We corrected the emission intensity by the temporal fluctuation of the laser power shown in the inset. **(E)** Dependence of the UC emission intensity on the simulated sunlight irradiance represented in units of sun (☉). The simulated sunlight comprised only wavelengths longer than 510 nm; see Enomoto et al., 2021 for details. In panels (C, E), we acquired the data represented by open marks by increasing the excitation power, and then we acquired the data represented by filled marks by decreasing the excitation power to check the quantitative reproducibility.

We prepared UC crystals from ANNP and PtOEP (Enomoto et al., 2021). Figure 3A (left panel) shows a photograph of the UC crystals actually used in this report. These crystals were transparent, were pinkish (uniformly colored; because of the PtOEP), and exhibited a thin flat-plate geometry. The volumetric concentration of the doped PtOEP in the crystal of ANNP was ca. 5.0 × 10^−5^ M, corresponding to a mole ratio of PtOEP to ANNP of approximately 1:50,000. The container in the photograph was a cylindrical quartz dish with an outer diameter and height of 17 and 5 mm, respectively.

Subsequently, we poured nondegassed pure water into the quartz dish and covered the dish with a quartz lid. For all experiments in this report, we used water from a commercial water purification system (Direct-Q UV 3, Merck Millipore). Because this water was purified at room temperature from tap water and had been stored under air in a tank until use, we assume that air was dissolved in it at almost the saturation concentration. We found that the crystals did not dissolve in water, which is reasonable considering the aromatic and thus hydrophobic nature of ANNP. Upon horizontal irradiation of continuous-wave laser light (wavelength: 542 nm, green–yellow) at the crystals immersed in water, we observed blue emission (Figure 3A, right panel).

In the following measurements (for Figures 3B–D), we characterized the UC emission properties of the crystals in nondegassed water held in a glass tube with a square cross section. This tube had inner and outer dimensions of 1 mm × 1 mm and 2 mm × 2 mm, respectively. To prevent evaporation of the water during the measurements, the open end of the glass tube was closed with a low melting point solder. Upon 542-nm laser light irradiation, we observed UC emission ranging from 425 to 500 nm (Figure 3B), coinciding with the fluorescence spectrum of ANNP (Enomoto et al., 2021). Notably, in the emission spectrum, we found no phosphorescence from PtOEP at ca. 650 nm, which would indicate solid aggregates of PtOEP (Goudarzi and Keivanidis, 2014; Karpicz et al., 2014; Vadrucci et al., 2014; Goudarzi and Keivanidis, 2017). Thus, the present material has resolved the long standing problem of sensitizer aggregation as we have discussed in detail (Enomoto et al., 2021).

Figure 3C shows the excitation intensity dependence of the sample crystals in nondegassed water. We first increased the intensity of the excitation at 542 nm (open marks) and then decreased it (filled marks) to check the reproducibility of the UC emission intensity. We fitted these data points to a theoretical curve [Eq. (36) of Murakami and Kamada, 2021]. From this fitting, we determined the excitation threshold intensity (*I*_th_) to be ca. 2.9 mW cm^−2^; which is sufficiently low for most *in vivo* photonic applications, considering that typical handheld laser pointers generate 1-mW optical power with a typical spot radius of 2–3 mm (i.e., 14–32 mW cm^−2^).

Figure 3D shows the results of our long-term stability tests of a sample under continuous photo-irradiation for >41 h in nondegassed water, measured at an excitation intensity of ca. 20 mW cm^−2^, ca. one order of magnitude greater than *I*_th_. We corrected the emission intensity by the fluctuation of the laser power as indicated in the inset. The stability of the UC emission, or that of the present material under continuous photo-irradiation, was excellent, even in the presence of oxygen in the water. This outstanding stability is attributable to the molecularly close-packed crystalline arrangement of ANNP as implied by the results of our previous single-crystal X-ray analysis (Enomoto et al., 2021). Because these UC properties are similar to those measured previously in air and the preparation procedure of the sample crystals (Figure 3A) was identical to that used beforehand, we hypothesize that the UC quantum efficiency here is similar to that determined previously in air (Enomoto et al., 2021). Such high photoirradiation stability in oxygen-dissolved water is highly advantageous for achieving broad types of aqueous photonic applications that can be rendered possible by utilizing UC.

Figure 3E shows dependence of the UC emission intensity on the simulated sunlight irradiance represented in units of Sun (☉), measured in nondegassed water; such a situation would be a typology for enhancing sunlight-driven photocatalysis in aqueous system with an assistance of UC. The simulated sunlight was passed through a 510 nm long-pass filter and thus comprised only wavelengths longer than 510 nm; see Enomoto et al., 2021 for experimental details. As shown by Figure 3E, the UC crystals exhibited low *I*_th_ of ca. 0.27☉, which is similar to *I*_th_ we reported previously in air (ca. 0.2☉; Enomoto et al., 2021). Therefore, the use of the present UC crystals in nondegassed water does not affect their superior *I*_th_.

We make some notes on our solid-solution crystals. So far, we have been able to make similar solid-solution crystals combining other sensitizers and ANNP, aiming to achieve a larger anti-Stokes shift. One representative case is a combination of ZnOEP and ANNP, which yielded light-yellowish crystals. ZnOEP in organic solvents typically exhibits Q-band absorption peaked at around 570 nm (which is longer wavelength than that of PtOEP). However, as indicated by Supplementary Figure S1A, the UC emission from the ZnOEP-ANNP solid-solution crystal is significantly weaker than that from the present PtOEP-ANNP solid-solution crystals. Our quantum-chemical simulations reveal that TET from ZnOEP to ANNP is highly unfavored (Supplementary Figure S1B). Thus, to achieve a large anti-Stokes shift, we have to find better annihilator that has a lower triplet energy level.

For some applications, i) solid-solution crystals formed on a flat surface or ii) solid-solution microcrystals may be useful. As for i), we recently reported a new method of forming a solid-solution polycrystalline film on a flat substrate (Enomoto and Murakami, 2023). However, this technique requires relatively low melting temperature for the annihilator (preferably lower than 150 °C). Thus, the high melting temperature of ANNP (254 °C) and resultant partial decomposition of ANNP have hindered the use of this new method for the present ANNP system, as shown by Supplementary Figure S2. As for ii), one of the easiest methods to form microcrystals is to use the rapid precipitation technique (*cf*. Figure 1C <ii>). However, the resulted PtOEP-doped-ANNP microcrystals exhibited rather poor performances (Section 3 of *Supplementary Material*; Supplementary Figure S3). This fact not only supports our discussion in Section 2 above but also corroborates the superiority of the near-equilibrium crystal growth to generate high quality solid-solution UC crystals. Therefore, to realize high-performance solid-solution UC microcrystals, a new way of nucleation density control under near-equilibrium crystal growth would be necessary.

## 5 Conclusion

This article has outlined the longstanding sensitizer aggregation problem and reviewed representative methods used to date for circumventing this problem via kinetic control. Whereas such kinetic approaches have progressed from initial works, the low crystallinity and small size of the crystalline domain that result from such fast solidification methods is also problematic. To fundamentally resolve this dilemma, we conceived and demonstrated the effectiveness of a solid-solution phase consisting of an α-phase in a bicomponent phase diagram (Figure 2A). In such van der Waals organic crystals prepared at much slower (*i.e.*, near-equilibrium) speeds, the driving force of the inclusion of a small quantity of one species (sensitizer) into a crystal of the other species (annihilator) is a thermodynamic force that results from the increased mixing entropy of two components (Schroeder, 2000).

Whereas such TTA–UC organic crystals have not been applied to aqueous systems beforehand, for the first time we have demonstrated that crystalline UC materials can function in a stable manner in water without degassing. The low excitation threshold and excellent photostability against continuous laser irradiation indicates that this material is promising for expanding the potential range of photo-related applications in aqueous environments; including *in vivo* diagnostic, clinical, and therapeutic applications.

## Data Availability

The raw data supporting the conclusion of this article will be made available by the authors, without undue reservation.
