# Supplementary material for: Stable and low-threshold photon upconversion in nondegassed water by organic crystals
Source: Front Chem. 2023 Jul 13;11:1217260. doi: 10.3389/fchem.2023.1217260 (PMC10373875; doi:10.3389/fchem.2023.1217260)
Supplement: Supplementary file 1 [file DataSheet1.pdf]

Supplementary Material

Stable and Low-Threshold Photon Upconversion in NonDegassed Water by Organic Crystals

Yoichi Murakami*, Riku Enomoto

Laboratory for Zero-Carbon Energy, Institute of Innovative Research, Tokyo Institute of Technology, 2-12-1 Ookayama, Meguro, Tokyo, Japan

*** Correspondence:** Yoichi Murakami: murakami.y.af@m.titech.ac.jp

Table of Contents

1. **Solid-Solution UC Crystals Prepared with ZnOEP**
2. **Differential Scanning Calorimetry (DSC) and Thermal Degradation of ANNP**
3. **Preparation and Characterizations of PtOEP-doped-ANNP Microcrystals**

1 Solid-Solution UC Crystals Prepared with ZnOEP

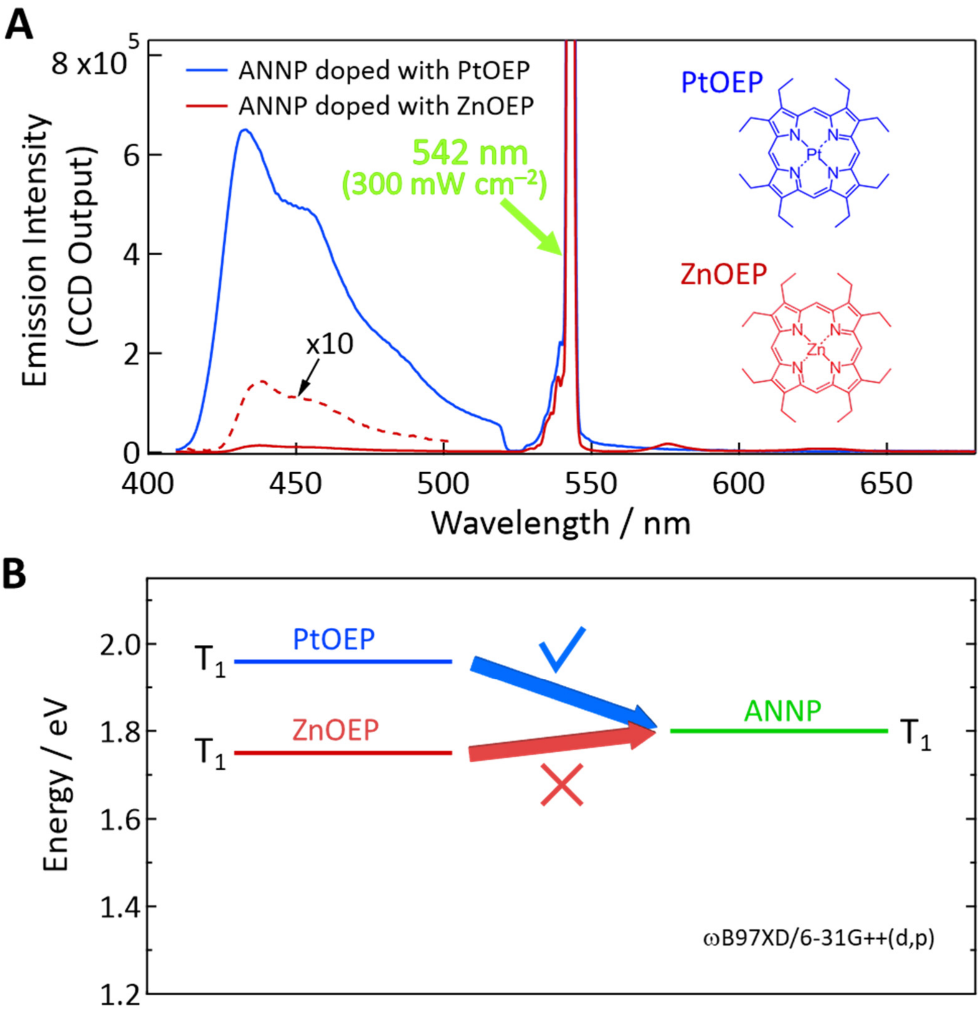

Supplementary Figure S1. (A) Photoemission spectra acquired using a 542-nm laser at 300 mW cm^{-1} for ANNP crystals doped with PtOEP (blue) and those dope with ZnOEP (red). **(B)** Energy level diagram indicating triplet-state energies of PtOEP (blue), ZnOEP (red), and ANNP (green). These energies were calculated using Gaussian 16® software with ωB97XD/6-31++G(d,p) level of theory. The results show that triplet energy transfer from ZnOEP to ANNP is highly unfavored.

2 Differential Scanning Calorimetry (DSC) and Thermal Degradation of ANNP

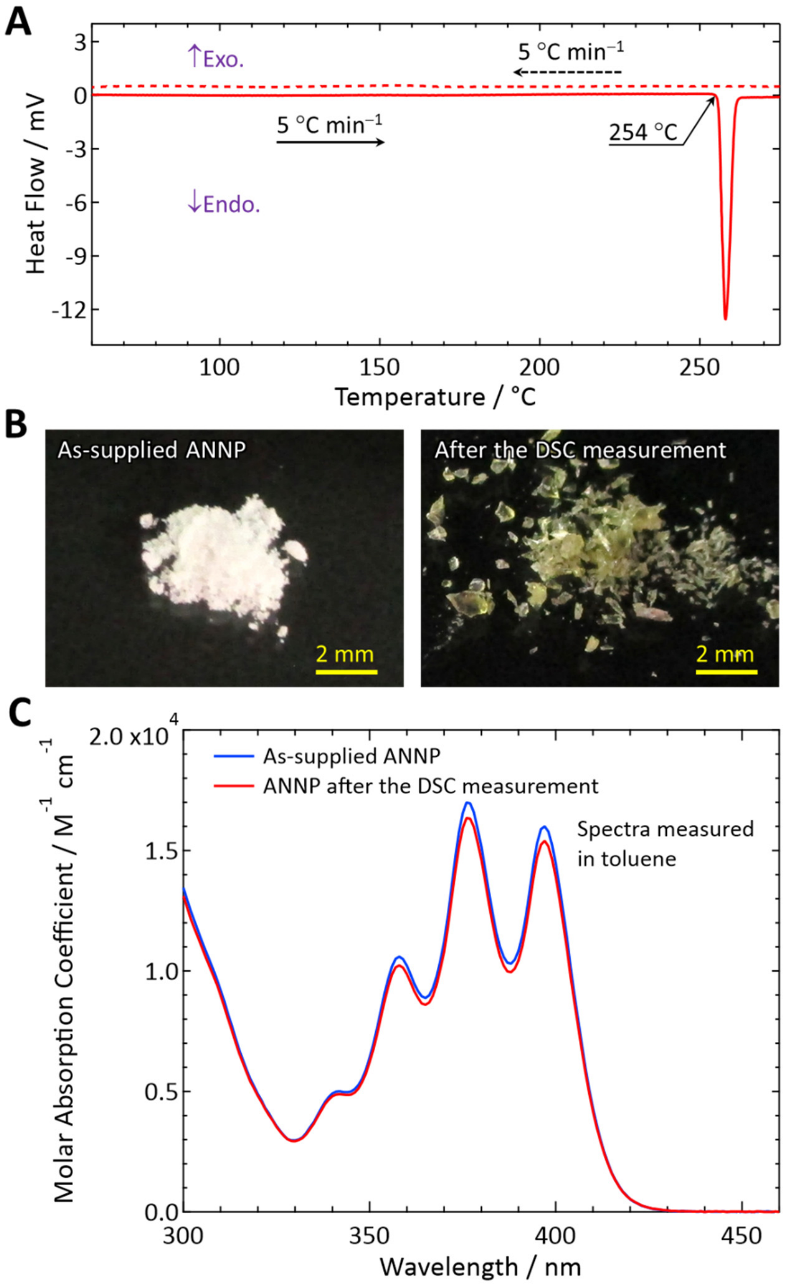

Supplementary Figure S2. Phase change behaviors and thermal degradation of ANNP. **(A)** Results of differential scanning calorimetry (DSC) of ANNP sealed in an aluminum crimp cell, conducted at a scan rate of 5 °C min^{-1} with flowing dry nitrogen gas. An endothermic change that started at 254 °C represents fusion of ANNP, whereas no clear exothermic change was found during the cooling process. This observation implies that the fusion has caused partial decomposition of ANNP. **(B)** Photographs of as-supplied ANNP powder (left) and ANNP after the DSC measurement (right). The latter indicated coloring as well as a sign of melting. **(C)** Comparison of the molar absorption coefficient spectra of as-supplied ANNP (blue) and that after the DSC measurement (red). The recognizable decrease of the molar absorption coefficient indicates partial decomposition of ANNP induced by the melting.

3 Preparation and Characterizations of PtOEP-doped-ANNP Microcrystals

We prepared PtOEP-doped-ANNP microcrystals by the following rapid precipitation method (see also Figure 1C <ii> in the main text). First, we prepared a toluene solution of PtOEP and ANNP (concentrations: 1.74×10^{-4} and 2.0×10^{-2} M, respectively). At room temperature, we poured this solution into ethanol so that the volume ratio of toluene to ethanol was 1:3. Ethanol is a poor solvent for PtOEP and ANNP. Immediately after this step, we ultrasonicated this mixture solution for 3 min; immediately after we started the ultrasonication, microcrystals were generated in the solution. Finally, the microcrystals were separated on a filter paper. Subsequently, we annealed these microcrystals according to the method reported in Enomoto et al., *Mater. Horiz.*, 2021, **8**, 3449–3456. The optical microscope images of the microcrystals after the annealing, the photoemission spectrum, and the excitation intensity dependence of the UC emission intensity are shown in Supplementary Figure S3 below. The color-sensitive polarizing microscope images taken rotating the sample stage indicate that they are actually crystalline. The performances were clearly poorer that those of our normal PtOEP-ANNP solid-solution UC crystals presented in the main text.

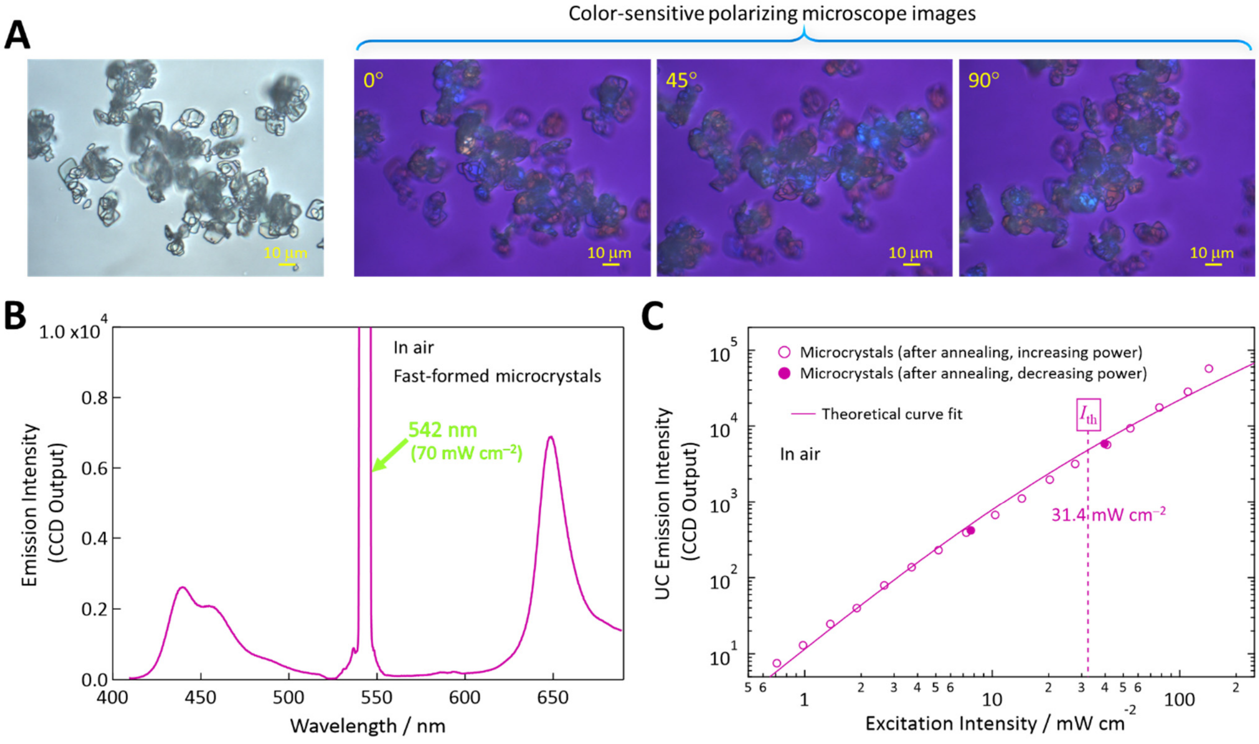

Supplementary Figure S3. (A) Optical microscope photographs of the prepared PtOEP-doped-ANNP microcrystals after the annealing. The color-sensitive polarizing microscope images were acquired under a crossed-Nicols configuration with a retardation plate of 530 nm. **(B)** Photoemission spectrum upon excitation of the microcrystals in air using a 542-nm laser at 70 mW cm^{-1}. The photoemission around 650 nm is phosphorescence from PtOEP. **(C)** Excitation intensity dependence of the UC emission intensity measured for the microcrystals using a 542-nm laser. The excitation threshold intensity (I_{th}) was found to be ca. 31 mW cm^{-1}.
